# Cytokine Output of Adipocyte-iNKT Cell Interplay Is Skewed by a Lipid-Rich Microenvironment

**DOI:** 10.3389/fendo.2020.00479

**Published:** 2020-07-31

**Authors:** Robert J. van Eijkeren, Imogen Morris, Anouska Borgman, Angela Markovska, Eric Kalkhoven

**Affiliations:** Molecular Cancer Research, Center for Molecular Medicine, University Medical Center Utrecht, Utrecht University, Utrecht, Netherlands

**Keywords:** adipocytes, iNKT cell, CD1d, lipolysis, insulin resistance

## Abstract

The complex direct and indirect interplay between adipocytes and various adipose tissue (AT)-resident immune cells plays an important role in maintaining local and whole-body insulin sensitivity. Adipocytes can directly interact with and activate AT-resident invariant natural killer T (iNKT) cells through CD1d-dependent presentation of lipid antigens, which is associated with anti-inflammatory cytokine production in lean AT (IL-4, IL-10). Whether alterations in the microenvironment, i.e., increased free fatty acids concentrations or altered cytokine/adipokine profiles as observed in obesity, directly affect adipocyte-iNKT cell communication and subsequent cytokine output is currently unknown. Here we show that the cytokine output of adipocyte-iNKT cell interplay is skewed by a lipid-rich microenvironment. Incubation of mature 3T3-L1 adipocytes with a mixture of saturated and unsaturated fatty acids specifically reduced insulin sensitivity and increased lipolysis. Reduced activation of the CD1d-invariant T-Cell Receptor (TCR) signaling axis was observed in Jurkat reporter cells expressing the invariant NKT TCR, while co-culture assays with a iNKT hybridoma cell line (DN32.D3) skewed the cytokine output toward reduced IL-4 secretion and increased IFNγ secretion. Importantly, co-culture assays of mature 3T3-L1 adipocytes with primary iNKT cells isolated from visceral AT showed a similar shift in cytokine output. Collectively, these data indicate that iNKT cells display considerable plasticity with respect to their cytokine output, which can be skewed toward a more pro-inflammatory profile *in vitro* by microenvironmental factors like fatty acids.

## Introduction

Insulin resistance is one of the hallmarks of type II diabetes mellitus (T2DM) pathogenesis, with both overlapping and unique molecular mechanisms affecting different metabolic organs, including muscle, liver and adipose tissue (AT) (1, 2). AT has long been thought of as a simple storage organ, however it has more recently been shown to be an extremely complex tissue which plays a key role in global homeostasis (3, 4). Important and intertwined mechanisms through which AT can communicate with other metabolic organs and cells are facilitated by the production of adipokines (5), which can act locally but also enter into the circulation. From here the AT adipokine action, both locally and systemically, acts to regulate immune behavior in specific cytokine output (3, 4, 6, 7). While the pathways leading from obesity to whole body insulin resistance and T2DM are complex and multifactorial (2), the increased uptake of nutrients leading to hyperplasia and hypertrophy of adipocytes is clearly an important early event, resulting in an altered adipokine secretion profile (5). In addition, hypertrophic AT displays a shift in AT-resident immune cells and cytokine output, where pro-inflammatory immune cells overwhelm the previously predominant anti-inflammatory immune cell populations (3, 6). The resulting chronic low-grade inflammation causes dysregulation of lipolysis, where adipocytes secrete higher levels of FFA, and glycerol (8, 9), which together with adipokines and cytokines can be viewed as an additional AT output. Combined these factors can have local or systemic effects and contribute to the development of whole-body insulin resistance and T2DM.

One of the AT-resident immune cell types that decrease dramatically with obesity both in mouse models and in humans are the invariant natural killer (iNKT) cells [reviewed in (10, 11)]. iNKT cells serve as a bridge between the innate and the adaptive immune system and are able to produce both pro-inflammatory cytokines, including IFNγ, and anti-inflammatory cytokines like IL-4 and IL-10 (10, 12, 13). IL-4 and IFNγ were initially thought to be on either end of the inflammatory spectrum, however recent research has shown that they also play a role in regulating other immune populations (14). Interestingly, the final steps of maturation of iNKT cells are thought to occur in the tissue where they reside, resulting in various tissue-defined subsets (10, 15). AT-resident iNKT cells for example have a Th2 cell phenotype and mainly produce anti-inflammatory cytokines IL-4 and IL-10 under lean conditions, which help to maintain AT homeostasis (16–20). iNKT cells are activated through a (semi)invariant TCR, that recognizes lipid antigens presented in the context of CD1d, a molecule that is expressed on the cell surface of various APC (13, 21, 22). In AT, iNKT cells can be directly activated by adipocytes, as they express not only CD1d itself but also possess a functional lipid antigen presentation pathway (16, 23–28), as well as a biosynthetic pathway for the production of lipid self-antigens (27, 28). Whilst in other biological settings the nature of lipid antigens, the type of APC and the microenvironment of the tissue have all been recognized as regulators of the secretion of Th1 and/or Th2 cytokines (10, 12, 29, 30), if and how the same parameters help to define AT-resident iNKT cell subsets and their cytokine output is largely unknown.

To study adipocyte-iNKT cell communication directly, i.e., without interference of other cell types, we and others have developed and characterized various co-culture assays, combining mouse or human (pre)adipocyte cell lines or primary adipocytes, with either reporter cells expressing the iNKT TCR (31), iNKT hybridoma cells (16, 24–28, 32) or primary iNKT cells isolated from AT or spleen (16, 24–28, 32). All these different assays strongly support direct adipocyte-iNKT cell communication, as they all show CD1d-dependent activation of the CD1d-iNKT TCR pathway upon co-culture with adipocytes, an activation boosted by the exogenous prototypical lipid antigen α-galactosylceramide (αGalCer), resulting in simultaneous production of both multiple pro- and anti-inflammatory cytokines (16, 24–28, 32).

Here we investigated the effects of FFA, as well as other obesity-associated components of the AT microenvironment, on the direct communication between adipocyte, and iNKT cells. First, we developed and characterized a cell culture model using mature murine 3T3-L1 adipocytes cell line treated with a commercially available and chemically-defined lipid mixture to mimic a high lipid microenvironment. Impaired insulin signaling and increased lipolysis was observed, without overall disruption of adipocyte-specific gene expression. Interestingly, while iNKT hybridoma cells, and primary iNKT cells produced both IL-4 and IFNγ under basal conditions, their cytokine output was skewed when adipocytes were pre-treated with the lipid mixture toward a low IL-4, high IFNγ profile. Taken together, these data indicate that iNKT cells display considerable plasticity with respect to their cytokine output, which can be skewed by microenvironmental factors like FFA.

## Materials and Methods

### Materials

Dexamethasone (Sigma), 3-isobutyl-1-methylxanthine (IBMX)(Sigma), insulin (I9278, Sigma), Lipid mix 1 (sigma L0288), albumin conjugated linoleic (sigma L9530), and oleic acid (sigma O3008), sodium palmitate (sigma P9767), myristic acid (sigma M3128), stearic acid (sigma S4751), cholesterol (MP biochemicals, 219934230), αGalactosylceramide KRN7000 (Avanti, 867000), IFNγ (sigma SRP3058), TNFα (sigma H8916), Pam3Cys (Calbiochem), LPS (sigma L4516), rabbit-anti-AKT (also named PKB) (33), rabbit-anti-pAKT-ser473 (4060S Cell Signaling).

### Cell Culture

The murine 3T3-L1 cell line (ZenBio) was cultured in Dulbecco's modified Eagle's medium (DMEM) supplemented with 10% bovine serum (Invitrogen), penicillin and streptomycin (both 100 μg/ml; Invitrogen). For differentiation of 3T3-L1 cells to adipocytes, the cells were grown to confluence and after 2 days (day 0) stimulated with culture medium containing dexamethasone (250 nM), 3-isobutyl-1-methylxanthine (500 μM), and insulin (170 nM) for 2 days. On day 2, the medium was changed for culture medium containing insulin (170 nM) and maintained for 4–6 days. The murine iNKT cell hybridoma line DN32.D3 was cultured in RPMI-1640 medium (Sigma Aldrich) supplemented with 10% fetal bovine serum, penicillin and streptomycin (both 100 μg/ml), MEM Non-Essential Amino Acids Solution (100x; ThermoFisher Scientific), HEPES (100x; Sigma Aldrich), Glutamine (100x, Sigma Aldrich) and βMeOH (100 μM; Sigma Aldrich). The JE6-1^REP−iNKT−β2M_KO^ reporter cell lines (31) were cultured in RPMI-1640 medium supplemented with 10% fetal bovine serum, penicillin and streptomycin (both 100 μg/ml).

Stimulation of 3T3-L1 adipocytes was done post differentiation for 3 days with lipid mix and individual fatty acids, and for 24 h with the residual obesogenic stimuli. One Millimolar of Palmitic, stearic and myristic acid was conjugated to fatty acid free BSA in a 6:1 ratio. Lipids were dissolved in 150 mM NaCl at 70°C while stirring. Before added to BSA 150 mM NaCl solution at 37°C while stirring. After adjusting pH to 7, 4 aliquots where stored at −20°C before further use (protocol adapted from Seahorse Bioscience).

### Western Blot Analysis

3T3-L1 cells were grown in a six well format and differentiated accordingly. After stimulation cells were washed with ice cold PBS, scraped and lysed in 300 μl ice cold RIPA buffer (150 mM NaCl, 1% NP40, 0.5% sodium DOC, 0.1 % SDS, 25 mM Tris pH 7.4, supplemented with protease inhibitor (Roche) and NaF acting as phosphorylase inhibitor) for 15′ at 4°C. After centrifugation on max speed at 4°C in table top centrifuge, supernatant was collected, supplemented with Laemmli Sample Buffer (LSB). All western blot samples were boiled for 5 min at 95°C before use. Samples were subjected to SDS-PAGE and transferred to PVDF membrane (Milipore). Blocking was done with 5% Skim Milk TBST. Primary antibody staining was performed overnight at 4°C (AKT & pAKT 1:2,000 in 5% BSA TBST). Secondary antibody staining was performed for 1 h at RT (1:1,000 in 5% milk TBST). After staining, membranes were washed for 1 h with TBST before being treated with ECL western blot substrate solution (Pierce). Protein expression was measured with LAS4000 ImageQuant.

### Glycerol Measurements

Secreted Glycerol was measured using the Sigma-aldrich Glycerol Assay Kit (#MAK117-1KT) with 50 μl of media.

### Triglyceride Measurements

Intracellular Triglycerides were measured using the kit Stanbio™ Triglycerides LiquiColor™ (#SB2200-225). Cells were washed with PBS and lysed in 50 μl cold PBS via syringe pull technique.

### Co-culture Assays

3T3-L1 wild type (Zenbio) were plated in a 96-well format and differentiated according to the protocol mentioned above. Mature 3T3-L1 Cells were then treated with lipid mix or individual fatty acids for 4 days, or 24 h with residual obesogenic stimulants. After stimulation, either DN32.D3 iNKT hybridoma cells (50.000 cells/well) or JE6-1^REP−iNKT−β2M_KO^ reporter cells (50.000 cells/well) were added to the 3T3-L1 adipocytes and co-cultured for 24 h. Medium from DN32.D3 co-cultures (200 μl) was used to determine secreted IL-4 and IFNγ via the Invitrogen™ eBioscience™ Mouse IL-4 ELISA Ready-SET-Go!™ Kit (#501128931) and the IFNγ ELISA kit (BD-bioscience).

JE6-1^REP−iNKT−β2M_KO^ reporter cells (31) were collected after co-culture by resuspending and collection in round bottom 96-well plates. Cells were then centrifuged (1,600 RPM, 5′, RT) and resuspended in 200 μl PBS. Cells were analyzed for eGFP expression by flow cytometry with FACSDiva (BD) and FlowJo (Tree Star Inc.) software. Data is presented as geometric Mean Fluorescent Intensity.

### RT-qPCR Analysis

RNA was extracted using TRIzol reagent (Invitrogen). cDNA was synthesized using the superscript first strand synthesis system (Invitrogen) according to manufacturer's protocol. Gene expression levels were determined by quantitative real time PCR with the MyIq cycler (Bio-Rad) using SYBR-green (Bio-Rad) and normalized to 36B4 expression. Primers for quantitative RT-PCR are described in Table S1.

### Single Cell Suspension From Spleen and Adipose Tissue (eWAT)

Tissue collection was performed on animals sacrificed for other purposes and therefore exempted form ethical approval by the local Animal Welfare Body Utrecht, a body of Utrecht University and the University Medical Center Utrecht (https://www.ivd-utrecht.nl/en/). Spleens and epididymal white AT (eWAT) were dissected from male C57BL/6J mice aged between 11 and 14 weeks. Spleens were disintegrated through a 70 μM cell strainer into 50 ml cold PBS and centrifuged for 10 min at 400 g. After discarding the supernatant, the pellet was resuspended in 5 ml of 10x Red Blood Cell (RBC) Lysis Buffer (Abcam; ab204733) for 5 min at room temperature. Forty five milliliter of cold PBS was added and cells centrifuged for 10 min at 400 g. After discarding the supernatant, the pellet was resuspended in 10 ml of cold PBS and filtered through a 70 μM cell strainer. Cells were retained on ice.

Before processing blood vessels and lymph nodes were removed from the eWAT which was then minced in 10 ml cold digestion buffer (Hanks' balanced salt solution with Ca^2+^ and Mg^2+^ supplemented with 0.5% bovine serum albumin). Per 1 g adipose, 1 ml of 10 mg/ml collagenase (Sigma-Aldrich; C6885) was added and incubated at 37°C for 15–20 min, with vigorous shaking every 5 min until AT was digested completely. Digested AT was then washed through a 100-μm cell filter with 20 ml of cold digestion buffer. Following centrifugation (500 g for 10 min) supernatant was removed and the cell pellet was resuspended in cold PBS. Cells were retained on ice.

### iNKT Purification

Cells isolated from spleen and adipose mice were pooled, respectively, before purification in order to remove individual variation and to maintain consistent cell numbers during co-culture. iNKT cells were purified from the pooled populations using the NK1.1+ iNKT Cell Isolation Kit, mouse (Miltenyi Biotec; #130-0960513). Purified cells were then used in co-culture as described above.

## Results

### Lipid Mixture Causes Insulin Insensitivity and Increased Lipolysis in Adipocytes

**Figure 1:**
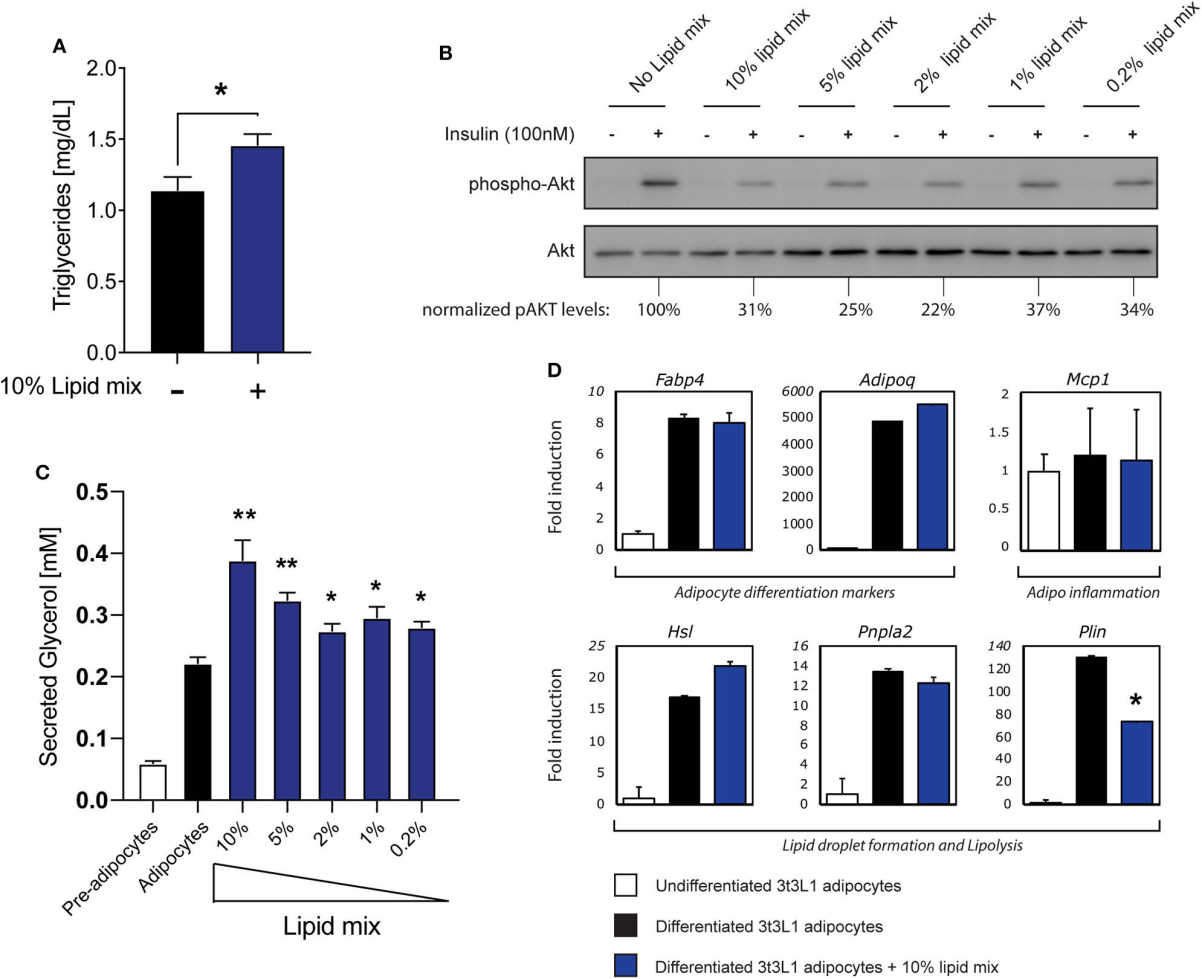
Stimulation with a lipid mixture causes an insulin resistance phenotype in 3T3-L1 adipocytes. **(A)** Mouse 3T3-L1 preadipocytes were differentiated into mature adipocytes and cultured with 10% lipid mixture for 4 days and intracellular triglyceride levels were determined. Statistical analysis via Students *t*-test against adipocytes glycerol secretion (NS *P* > 0.05, ^*^P < 0.05, ^**^P < 0.01, ^***^P < 0.001) (*n* = 3). **(B)** Mature 3T3-L1 adipocytes were treated with different concentrations (10–0.2%) Lipid Mixture for 4 days and deprived of insulin signaling before insulin stimulation (30 min). Lysates subjected to Western blot analysis. Western blot quantification of phospho-AKT (ser473) normalized to total AKT levels. **(C)** Quantification of glycerol secreted by 3T3-L1 adipocytes, and control pre-adipocytes, after 4 days in culture with titrated lipid mix dilutions. Statistical analysis via Students *t*-test against adipocyte glycerol secretion (NS *P* > 0.05, ^*^P < 0.05, ^**^P < 0.01) (*n* = 3). **(D)** Mouse 3T3-L1 preadipocytes were differentiated into mature adipocytes and treated with 10% lipid mix for 4 days before being subjected to RNA isolation and quantitative RT PCR. Transcriptional activity of genes involved in adipogenesis, inflammation and lipolysis are depicted as fold induction relative to undifferentiated 3T3-L1 adipocytes. Data are normalized to housekeeping gene 36B4 and presented as mean +/– SD (*n* = 6).

The increased concentration of circulating FFA observed in obesity contributes to the development of insulin resistance in adipose tissue (1, 2). To mimic this phenomenon in a cell culture model we used a chemically defined, commercially available lipid mixture containing cholesterol plus monounsaturated FFA (oleic acid), polyunsaturated FFA (arachidonic, linolenic, and linoleic acid) and saturated FFA (myristic, palmitic, and stearic acid), as reported previously (34). A significant increase in triglyceride (TG) storage was observed in mature 3T3-L1 adipocytes after culturing with 10% lipid mixture (Figure 1A), Next, the effects of different concentrations of lipid mixture on insulin sensitivity was assessed by re-stimulating insulin deprived mature 3T3-L1 adipocytes cultured in the absence or presence of various concentrations of lipid mixture. Insulin signaling decreased 3–4-fold compared to untreated adipocytes, with 10% lipid mixture displaying the maximal effect (Figure 1B). When tested individually, the inhibitory effect on insulin signaling by the different components of the lipid mixture was not observed when compared to the lipid mixture (Figure S1A). In addition, no inhibitory effect was observed after stimulation with cytokines TNFα and IFNγ (Figure S1A), which are both elevated in obesity (35, 36). To analyse functional effects of the reduced insulin signaling, we focused on lipolysis, a process that is highly regulated by insulin (2). As shown in Figure 1C a significant increase in lipolysis was observed when cells were subjected to the lipid mixture, as assessed by analyzing secreted glycerol concentrations. Again, stimulation with the individual components of the lipid mixture did not increase glycerol secretion significantly (Figure S1B). Additionally, stimulation with cytokines TNFα and IFNγ increases glycerol secretion (Figure S1C) but similar stimulation showed no effect on insulin signaling (Figure S1A). Also, activation of TLR2 (Pam3Cys) or TLR4 (LPS), which has been shown to occur in obesity (37–39), has no outspoken effect on glycerol secretion (Figure S1C).

To characterize this cell model further, we analyzed the effects of the lipid mixture on mRNA expression of genes associated with adipocyte differentiation (adipogenesis), inflammation, and lipid storage by RT-qPCR (Figure 1D). Expression of the adipogenesis markers FABP4 and Adipoq (encoding adiponectin) were clearly upregulated during adipogenesis with expression remaining consistent irrespective of Lipid Mix (Figure 1D). The inflammation marker gene *Mcp1* was expressed in both undifferentiated and differentiated cells, and unaffected by the lipid mixture (Figure 1D). Furthermore, as we observed increased triglyceride storage and glycerol secretion in mature 3T3-L1 adipocytes subjected to the lipid mixture (Figures 1A,C), we analyzed the expression of genes involved in lipid storage and lipolysis. *HSL* and *PNPLA2* (encoding the ATGL protein) were more highly expressed in differentiated cells compared to undifferentiated 3T3-L1 cells, but no significant change in expression was observed upon lipid mixture treatment. The *Plin* gene, encoding Perilipin1, was the only gene tested here to show a significant decrease in expression. As also observed for insulin signaling (Figure S1A) and lipolysis (Figure S1B), none of the lipolysis related genes we tested increased upon stimulation with the main individual components that make up the lipid mix (Figure S1D). To verify that expression of the genes analyzed in general was not static but could be modified by other stimuli, we subjected the cells to other obesity-associated stimuli (TNFα, IFNγ) or inflammatory stimuli (Pam3Cys, LPS) and observed various changes in gene expression (Figure S1E).

Taken together, these data indicate that treatment of mature 3T3-L1 adipocytes with a chemically-defined lipid mixture results in a robust insulin resistance phenotype with increased lipolysis, without causing an overall disruption of cellular functionality, as the cells were clearly functional in terms of lipid metabolism and no dramatic changes in various key genes were observed. These observed characteristics support this cellular model as suitable for subsequent experimental approaches, including *in vitro* studies on adipocyte-iNKT cell communication.

### Lipid Mixture Skews Cytokine Output in Adipocyte-iNKT Interplay

**Figure 2:**
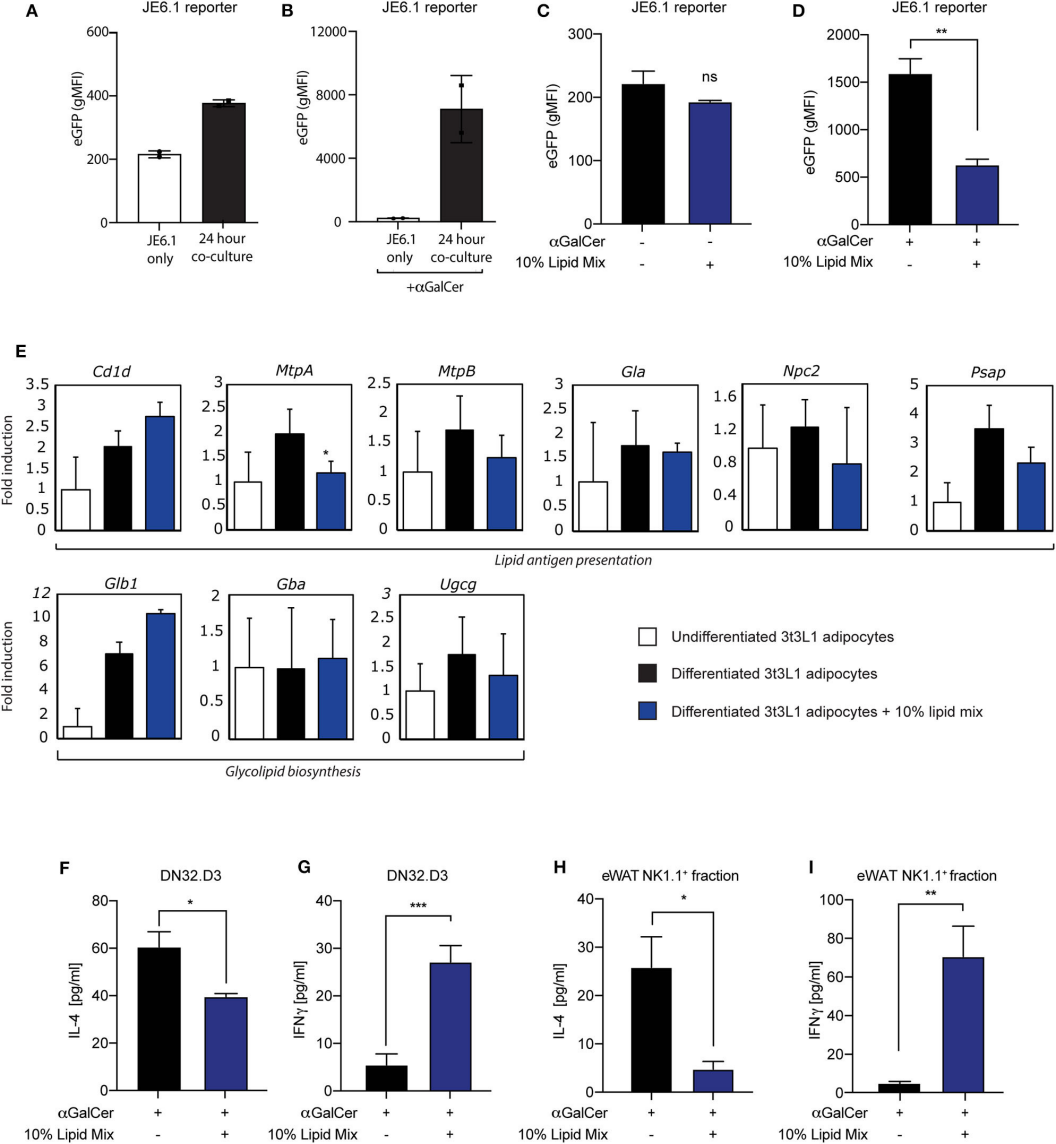
Lipid mixture treatment skews iNKT cell cytokine secretion toward a pro-inflammatory profile after co-culture. **(A,B)** Co-culture between JE6-1^REP−iNKT−β2M_KO^ reporter cells and mature 3T3-L1 adipocytes stimulated with **(B)** or without **(A)** αGalCer (0.5 μg/ml) 24 h prior to co-culture (*n* = 2) Data is presented as geometric Mean Fluorescent Intensity (gMFI). **(C,D)** Co-culture between JE6-1^REP−iNKT−β2M_KO^ reporter cell (5 × 10^4^ cells per well in a 96 well-format) and mature 3T3-L1 adipocytes treated with or without 10% lipid mix and stimulated with **(D)** or without **(C)** αGalCer (0.5 μg/ml). JE6-1^REP−iNKT−β2M_KO^ cells were collected after 24 h co-culture and analyzed for GFP expression by FACS. Data is presented as geometric Mean Fluorescent Intensity (gMFI) +/− SD (*n* = 3, *P* = 0.0044). **(E)** Mouse 3T3-L1 preadipocytes were differentiated into mature adipocytes and treated with 10% lipid mix for 4 days before being subjected to RNA isolations and Quantitative RT PCR. Transcriptional activity of genes involved in adipogenesis, glycolipid biosynthesis, lipid antigen presentation, and lipolysis are depicted as fold induction relative to undifferentiated 3T3-L1 adipocytes. Data is normalized to housekeeping gene 36B4 and presented as mean +/− SD (*n* = 6). **(F,G)** ELISA analysis of IL-4 and IFNγ secreted by DN32.D3 cells after 24 h co-culture with mature 3T3-L1 adipocytes treated with or without 10% lipid mix (*n* = 3, *p* = 0.0319 and 0.0004 for IL-4 & IFNγ, respectively) Each data point represents 5 × 10^4^ cells. **(H)** IL-4 secretion following 24 h co-culture of NK1.1 positive *ex-vivo* fraction extracted from visceral eWAT, cultured with αGalCer stimulated (0.5 μg/ml) mature 3T3-L1 adipocytes treated with and without 10% lipid mix. Each data point represents 5 × 10^4^ cells taken from a pooled population, statistical analysis via Students *t*-test against (NS *P* > 0.05, ^*^P < 0.05, ^**^P < 0.01, ^***^P < 0.001) (*n* = 3) **(I)** IFNγ secretion following 24 h co-culture of NK1.1 positive *ex-vivo* fraction extracted from eWAT, cultured with αGalCer stimulated (0.5 μg/ml) mature 3T3-L1 adipocytes treated with and without 10% lipid mixture. Each data point represents 5 × 10^4^ cells taken from a pooled population. Statistical analysis via Students *t*-test against (NS *P* > 0.05, ^*^P < 0.05, ^**^P < 0.01, ^***^P < 0.001) (*n* = 3–6).

Having established a cellular adipocyte model with a high-lipid microenvironment (Figure 1), we next wished to investigate if and how adipocyte-iNKT cell communication is altered under these experimental conditions. For this we used different experimental co-culture approaches. First, we cultured mature 3T3-L1 adipocytes pre-treated with lipid mixture or left untreated together with the recently developed JE6-1^REP−iNKT−β2M_KO^ reporter cells (31). This reporter cell line is based on the Jurkat T cell line, stably transfected with an NFkB-eGFP reporter and the human Vα24-Jα18 TCRα chain and Vβ11 TCRβ chain (31). Co-culture of lipid antigen presenting cells followed by quantification by FACS analysis provides a sensitive fluorescence-based readout of iNKT TCR-lipid antigen interaction (31). In addition, the β2M gene was deleted from these cells using CRISPR/Cas9, to eliminate antigen-selfpresentation and self-activation (31). When JE6-1^REP−iNKT−β2M_KO^ reporters were cultured for 24 h with lipid mixture- treated mature 3T3-L1 adipocytes, an increase in eGFP expression was observed compared to individually cultured reporter cells, which was boosted by the prototypical lipid antigen αGalCer (Figures 2A,B). These results expand the use of these reporter cells as a read-out for CD1d-iNKT TCR signaling to adipocytes (31). Furthermore, as similar observations were previously made using iNKT hybridoma cells (16, 24–28, 32), we conclude that these reporter cells provide a robust read-out system for 3T3-L1 adipocyte-iNKT cell interaction. When using this read-out system to investigate the effect of lipid mixture treatment, eGFP expression decreased when adipocytes were stimulated with αGalCer 24 h prior to co-culture (Figure 2D), a trend not observed in the absence of αGalCer (Figure 2C). Importantly, the lipid mixture or individual components did not alter eGFP expression when the reporter cells were tested in isolation, indicating that CD1d-iNKT TCR signaling is required (Figure S2B). Also, individual components of the lipid mix and the inflammatory stimuli IFNγ, Pam3Cys, and LPS did not alter eGFP expression after co-culture significantly (Figures S2C–F). It should be noted that TNFα did induce eGFP expression, as it can activate the NFkB reporter present in the JE6-1^REP−iNKT−β2M_KO^ reporter cells (Figure S2A).

To investigate potential mechanisms behind this change in adipocyte-iNKT reporter cell communication, we analyzed the expression of several genes that have previously been implicated in lipid antigen presentation in adipocytes (*Cd1d, MtpA*, and *MtpB*), or that could potentially play in role in this based on findings in other lipid APC (*Gla, Npc2, Psap*). In addition, we analyzed 3 genes implicated in the biosynthesis of potential endogenous lipid antigens in adipocytes (*Ugcg*) and other lipid APC's (*Glb1, Gba*). In agreement with previous reports (26, 27, 32), the *Cd1d* gene, encoding the actual lipid antigen presenting molecule, was upregulated during 3T3-L1 differentiation, here no effect of the lipid mixture was observed (Figure 2E). Also, expression of the 2 isoforms of Microsomal Triglyceride Transfer Protein [*MtpA* and *MtpB;* note that *MtpB* is predominantly expressed in 3T3-L1 adipocytes (27)], a factor we previously implicated in lipid antigen presentation in adipocytes (27), displayed <2-fold change. The expression of other lipid antigen loading machinery genes which potentially play a role in lipid antigen presentation in adipocytes (27) like pro-saposin (*Psap*), Niemann Pick type C2 (*Npc2*), α-galactosidase (*Gla*), were also not altered dramatically (Figure 2E). The *Ugcg* gene, encoding ceramide glucosyltransferase and implicated in the biosynthesis of endogenous lipid antigens in adipocytes and other APC (28, 40), also displayed a limited change upon lipid mixture treatment (Figure 2E), as did two other genes potentially involved in the biosynthesis of endogenous lipid antigens, *Glb1*, encoding β-galactosidase, and *Gba*, encoding beta-glucocerebrosidase (Figure 2E). Stimulation with individual lipid mix components also shows no dramatic differences in gene expression of lipid antigen presentation and glycolipid biosynthesis genes (Figure S1D). Taken together, these data indicate that a lipid-rich microenvironment can alter communication through the CD1d-iNKT TCR axis, but that changes in genes implicated in lipid antigen presentation are not likely to present the underlying mechanism.

The second read-out system used was co-culture of mature 3T3-L1 adipocytes with mouse iNKT hybridoma cells followed by cytokine analysis, a robust system previously used by us and others (26–28, 32). In line with the JE6-1^REP−iNKT−β2M_KO^ reporter-based system (Figure 2D), we saw a significant decrease in secretion of the anti-inflammatory cytokine IL-4 by the iNKT cell hybridoma DN32.D3 when adipocytes were subjected to the lipid mixture (Figure 2F). In contrast, a significant increase in secretion of the pro-inflammatory cytokine IFNγ was observed upon lipid mixture treatment (Figure 2G), indicating plasticity of the iNKT cell in terms of cytokine output depending on the microenvironment. Again, in line with the JE6-1^REP−iNKT−β2M_KO^ reporter-based system, individual components of the lipid mix and inflammatory stimuli did not alter cytokine secretion by DN32.D3 iNKT cells in a co-culture setting (Figures S2G,H). Also, 3T3-L1 adipocytes treated with 10% lipid mixture did not produce high levels of IFNγ that may conflict with the outcome of 3T3-L1-DN32.D3 co-cultures (Figure S2I).

Whilst the JE6-1^REP−iNKT−β2M_KO^ reporter cells and DN32.D3 iNKT cell hybridoma represent useful proxies for iNKT cells they do not fully reflect all iNKT cell characteristics, which are in part tissue-specific (10). Therefore, as a third read-out system we replaced the DN32.D3 hybridoma with primary iNKT cells isolated from either spleen or eWAT, based on the NK1.1 marker (NK1.1.+ iNKT cells) as reported previously (16). Similar to the DN32.D3 based co-culture system (Figures 2F,G), NK1.1^+^ eWAT iNKT cells co-cultured with mature 3T3-L1 adipocytes treated with lipid mixture show a significant reduction (*p* = 0.0233) in IL-4 (Figure 2H) and an significant increase (*p* = 0.0086) in IFNγ secretion (Figure 2I). On the other hand, the response of NK1.1^+^ iNKT cells isolated from mouse spleens was far less pronounced for both IL-4 and IFNγ secretion (Figures S3A,B). Furthermore, the NK1.1^−^ fraction from both eWAT and spleen followed the same trend as their positive selection counterparts, but to a lower extent (Figures S3C,D). These data indicate that the eWAT iNKT cytokine secretion profile has a specific plasticity and is highly responsive to the adipose environment, with a key role for free fatty acids in this process.

## Discussion

Aside from their CD1d restricted TCR, iNKT cells can have divergent functions, surface markers and cytokine secretion preferences, all of which seem to be determined by the nature of lipid antigens, the type of APC and the microenvironment of the tissue (21, 41, 42). Despite their environmental priming, they also appear to retain plasticity, enabling them to respond swiftly and dynamically to changes in their surroundings (10, 12, 29, 43). Given the complex nature of AT and the various interactions between the different cell types present in this tissue, we decided to develop assay systems where it would be possible to study adipose—iNKT crosstalk in isolation. To facilitate this, we optimized a lipid enriched adipose cell line 3T3-L1 which exhibits several obesity-associated characteristics without disturbing functionality (Figure 1E). Using two iNKT model cell lines, DN32.D3, and JE6-1^REP−iNKT−β2M_KO^ reporter cells, we show that cross-talk with the lipid enriched 3T3-L1 adipocytes influences output following TCR stimulation (Figure 2). Interestingly, iNKT cells extracted from eWAT, like DN32.D3 hybridoma cells, display an IFNγ secretion skew over IL-4 in lipid rich environments. On the other hand, splenic iNKT cells do not show the same capacity in this context, highlighting the divergence between the two populations. Based on this rapid response to a lipid rich environment, we conclude that eWAT iNKT cells harbor significant plasticity in terms of their cytokine output, which is pre-primed by their tissue micro-environment. Interestingly, a short term HFD diet intervention in which skewing of cytokine output by AT-resident iNKT cells was reported by Li et al. (20). It should be noted however that while we observed a skew toward IFNγ production upon treatment with a defined lipid mixture, Li et al. (20) reported skewing toward higher IL-4 production in AT upon HFD feeding. Although further research is needed to establish how the *in vivo* and *in vitro* interventions can be translated into each other, both studies support the view that the regulatory function of iNKT cells is dynamic and complex, exemplified by their ability to rapidly switch their cytokine preference based on their micro-environment.

In the present study we compared several co-culture systems to address the delicate cross-talk between adipose and iNKT cells in a lipid-rich environment. Under our experimental conditions, we obtained similar results from both DN32.D3 hybridoma cells and primary iNKT cells form eWAT, suggesting that DN32.D3 hybridoma cells represent an appropriate model for iNKT cells in the context of eWAT (Figure 2). In our co-culture assays, the response of the JE6-1^REP−iNKT−β2M_KO^ reporter cells (31) reflected the effects observed in the other systems on IL-4 secretion, i.e., reduced output when adipocytes were pre-treated with lipid mixture, but not the increased IFNγ secretion. It should be noted that the NFkB-eGFP reporter in the JE6-1^REP−iNKT−β2M_KO^ reporter cells provides a single read-out (31); our data suggest that the intracellular pathways ultimately leading to IFNγ and IL-4 secretion are wired differently at some level, but the underlying molecular mechanisms remain to be established. The JE6-1^REP−iNKT−β2M_KO^ reporter cells nonetheless clearly present a very valuable tool for identification and verification of (endogenous) lipid antigens (31).

In contrast to iNKT cells from eWAT, we have also shown in the present study that splenic iNKT cells do not exhibit an IL-4/IFNγ preference in co-culture assays upon lipid mixture treatment. Splenic iNKT cells have been previously reported to have an IL-4 and IFNγ cytokine secretion capacity (10, 43), we show that this capacity is not significantly altered by lipid enrichment. As previously stated, we focused on IL-4 and IFNγ because of their duel nature, as both are linked to inflammatory balance as well as their regulatory implications for the surrounding immune populations (44–49). Therefore, the contrast between AT and splenic iNKT populations emphasizes that isolating immune cells from the tissue that is being studied for subsequent analyses is recommendable. This is particularly true for iNKT cells, as iNKT populations for the most remain in one tissue environment with only few cells in circulation, in contrast to for example the much more mobile macrophages (17, 41, 43, 46).

Immune cell plasticity refers to immune cells which have a flexible, context-dependent inflammatory phenotype. This flexibility allows for rapid response to stimulus without being permanently defined by it (41, 43, 48, 50). We can draw parallels with other more defined immune populations. A good example of this is M1/M2 macrophages, where the macrophage population is capable of being on either end of the inflammatory spectrum and anywhere in between (19, 48, 49, 51). Similarly, iNKT cells are able to secrete a multitude of cytokines but in a context dependent pre-primed manner (6, 10, 14, 41, 52–54). Therefore, we propose that iNKT cells have a plasticity in response to their surroundings, and that their response is potentially tailored to the specific requirements of their micro-environment. How the plasticity observed in our proof of principle studies translates into the complex interplay between adipocytes and various AT-resident immune cell types *in vivo*, and ultimately into whole body energy homeostasis will be the topic of future studies. Indeed, very recent *in vivo* studies by LaMarche et al. already underscored the microenvironmental impact on iNKT cell output (55).

## Data Availability Statement

All datasets generated for this study are included in the article/Supplementary Material.

## Ethics Statement

Ethical review and approval was not required for the animal study because it was exempted by the local animal ethics committee.

## Author Contributions

RE, IM, AB, AM, and EK designed the experiments. RE, IM, AB, and AM performed experiments and analyzed the data. RE and IM drafted the manuscript. RE, IM, and EK edited and revised the manuscript. All authors contributed to the article and approved the submitted version.

## Conflict of Interest

The authors declare that the research was conducted in the absence of any commercial or financial relationships that could be construed as a potential conflict of interest.
