# Supplementary material for: Cytokine Output of Adipocyte-iNKT Cell Interplay Is Skewed by a Lipid-Rich Microenvironment
Source: Front Endocrinol (Lausanne). 2020 Jul 31;11:479. doi: 10.3389/fendo.2020.00479 (PMC7412741; doi:10.3389/fendo.2020.00479)
Supplement: Supplementary file 1 [file Data_Sheet_1.pdf]

Cytokine output of adipocyte-iNKT cell interplay is skewed by a lipid-rich microenvironment

Robert J. van Eijkeren[1#], Imogen Morris [1#], Anouska Borgman[1] Angela Markovska[1], and Eric Kalkhoven[1,*]

[1] Molecular Cancer Research, Center for Molecular Medicine, University Medical Center Utrecht, Utrecht University, Utrecht the Netherlands.

[#] Authors contributed equally

[*]Correspondence:

Dr. Eric Kalkhoven

e.kalkhoven@umcutrecht.nl

Author contribution statement

R.J.v.E., I.M., A.B., A.M. and E.K. designed the experiments; R.J.v.E., I.M., A.B. and A.M. performed experiments and analysed the data; R.J.v.E. and I.M. drafted the manuscript; R.J.v.E., I.M. and E.K. edited and revised the manuscript; all authors approved the final version of the manuscript.

Supplementary data

Figure S1, (A) Mature 3T3-L1 adipocytes were treated with 10% Lipid Mixture, oleic acid (30μM), linoleic acid (30μM), palmitic acid (30μM), IFNγ (20μg/ml) or TNFα (4ng/ml) for 4 days after differentiation and deprived of insulin (24 hours in 0.5% serum DMEM) before insulin stimulation (100nM, 30 minutes). Lysates were subjected to Western blot analysis for AKT and phospho-AKT (ser473) expression. (B) Secreted glycerol measurement on medium derived from mature 3T3-L1 adipocytes treated with individual components of lipid mix (30μM) (n = 3). (C) Secreted glycerol measurement on medium derived from mature 3T3-L1 adipocytes treated with different inflammatory stimuli (20μg/ml IFNγ, 1μg/ml LPS, 2μg/ml Pam3Cys, 4 ng/ml TNFα) (n = 3). (D) RNA was isolated from mature 3T3-L1 adipocytes treated with individual components of lipid mix (30μM) for four days and subjected to Quantitative RT PCR analysis. Transcriptional activity of genes involved in adipogenesis, inflammation, glycolipid biosynthesis, lipid antigen presentation and lipolysis are depicted as fold induction relative to undifferentiated 3T3-L1 adipocytes. Data are normalized to housekeeping gene 36B4 and presented as mean +/- SD (n = 6) (E) RNA was isolated from mature 3T3-L1 adipocytes treated with different inflammatory stimuli (20μg/ml IFNγ, 1μg/ml LPS, 2μg/ml Pam3Cys, 4 ng/ml TNFα) and subjected to Quantitative RT PCR analysis Transcriptional activity of genes involved in adipogenesis, inflammation, glycolipid biosynthesis, lipid antigen presentation and lipolysis are depicted as fold induction relative to undifferentiated 3T3-L1 adipocytes. Data are normalized to housekeeping gene 36B4 and presented as mean +/- SD (n = 6).

*Figure S2 (*A) Gating strategy based on unstimulated JE6-1$^{REP\text{-}iNKT\text{-}\beta2M_KO}$ cells (negative control, upper lane) and iNKT reporter cells stimulated with TNFα (positive control, lower lane), both without prior co-culture with adipocytes. (B) JE6-1$^{REP\text{-}iNKT\text{-}\beta2M_KO}$ reporter cells treated with different concentrations lipid mix, individual lipids and inflammatory stimuli and stimulated with or without αGalCer (0.5 μg/ml). JE6-1$^{REP\text{-}iNKT\text{-}\beta2M_KO}$ cells were collected after 24hr culture and analysed for GFP expression by FACS. Data is presented as geometric Mean Fluorescent Intensity (gMFI) +/- SD (N=3, 2 & 1). (C, D) Co-culture between JE6-1$^{REP\text{-}iNKT\text{-}\beta2M_KO}$ reporter cells and mature 3T3-L1 adipocytes treated with different fatty acids (30μM) for 4 days and 24 hr with (D) or without (C) αGalCer (0.5 μg/ml). JE6-1$^{REP\text{-}iNKT\text{-}\beta2M_KO}$ cells were collected after 24hr co-culture and analysed for GFP expression by FACS. Data is presented as geometric Mean Fluorescent Intensity (gMFI) +/- SD (n = 3). (E, F) Co-culture between JE6-1$^{REP\text{-}iNKT\text{-}\beta2M_KO}$ reporter cells and mature 3T3-L1 adipocytes treated with different inflammatory stimuli (20μg/ml IFNγ, 1μg/ml LPS, 2μg/ml Pam3Cys) and stimulated with (F) or without (E) αGalCer (0.5 μg/ml). JE6-1$^{REP\text{-}iNKT\text{-}\beta2M_KO}$ cells were collected after 24hr co-culture and analysed for GFP expression by FACS. Data is presented as geometric Mean Fluorescent Intensity (gMFI) +/- SD (n = 3). (G/H) ELISA analysis of IL-4 and IFNγ secreted by DN32.D3 cells after 24 hour co-culture with mature 3T3-L1 adipocytes treated different inflammatory stimuli (20μg/ml IFNγ, 1μg/ml LPS, 2μg/ml Pam3Cys, 4 ng/ml TNFα) stimulated with (H) or without (G) αGalCer (0.5 μg/ml). Each data point represents 50.000 cells. (i) ELISA analysis of IFNγ on medium directly or medium collected from 3T3L1 cells treated with 10% lipid mixture for 4 days (n=3).

Figure S3 (A) IL4 secretion following 24h co-culture of NK1.1 positive ex-vivo fraction extracted from the spleen, cultured with αGalCer stimulated (0.5 μg/ml) mature 3T3-L1 adipocytes treated with and without 10% lipid mix. Each data point represents 50.000 cells taken from a pooled population, statistical analysis via Students t-test against (ns $P > 0.05$, * $P < 0.05$, ** $P < 0.01$, *** $P < 0.001$) ($n = 3$) (B) IFNγ secretion following 24h co-culture of NK1.1 positive ex-vivo fraction extracted from the spleen, cultured with αGalCer stimulated (0.5 μg/ml) mature 3T3-L1 adipocytes treated with and without 10% lipid mix. Each data point represents 50.000 cells taken from a pooled population. Statistical analysis via Students t-test against (NS $P > 0.05$, * $P < 0.05$, ** $P < 0.01$, *** $P < 0.001$) ($n = 3 – 6$). (C) IL-4 secretion following 24h co-culture of NK1.1 negative ex-vivo fraction extracted from the spleen or eWAT, cultured with adipocytes with and without 10% lipid mix. Each data point represents 50.000 cells taken from a pooled population. Statistical analysis via Students t-test against (NS $P > 0.05$, * $P < 0.05$, ** $P < 0.01$, *** $P < 0.001$) ($n = 3 – 8$). (D) IFNγ secretion following 24h co-culture of NK1.1 negative ex-vivo fraction extracted from the spleen or eWAT cultured with adipocytes with and without 10% lipid mix. Each data point represents 50.000 cells taken from a pooled population. Statistical analysis via Students t-test against (NS $P > 0.05$, * $P < 0.05$, ** $P < 0.01$, *** $P < 0.001$) ($n = 3 – 8$).

SUPPLEMENTARY FIGURE 1

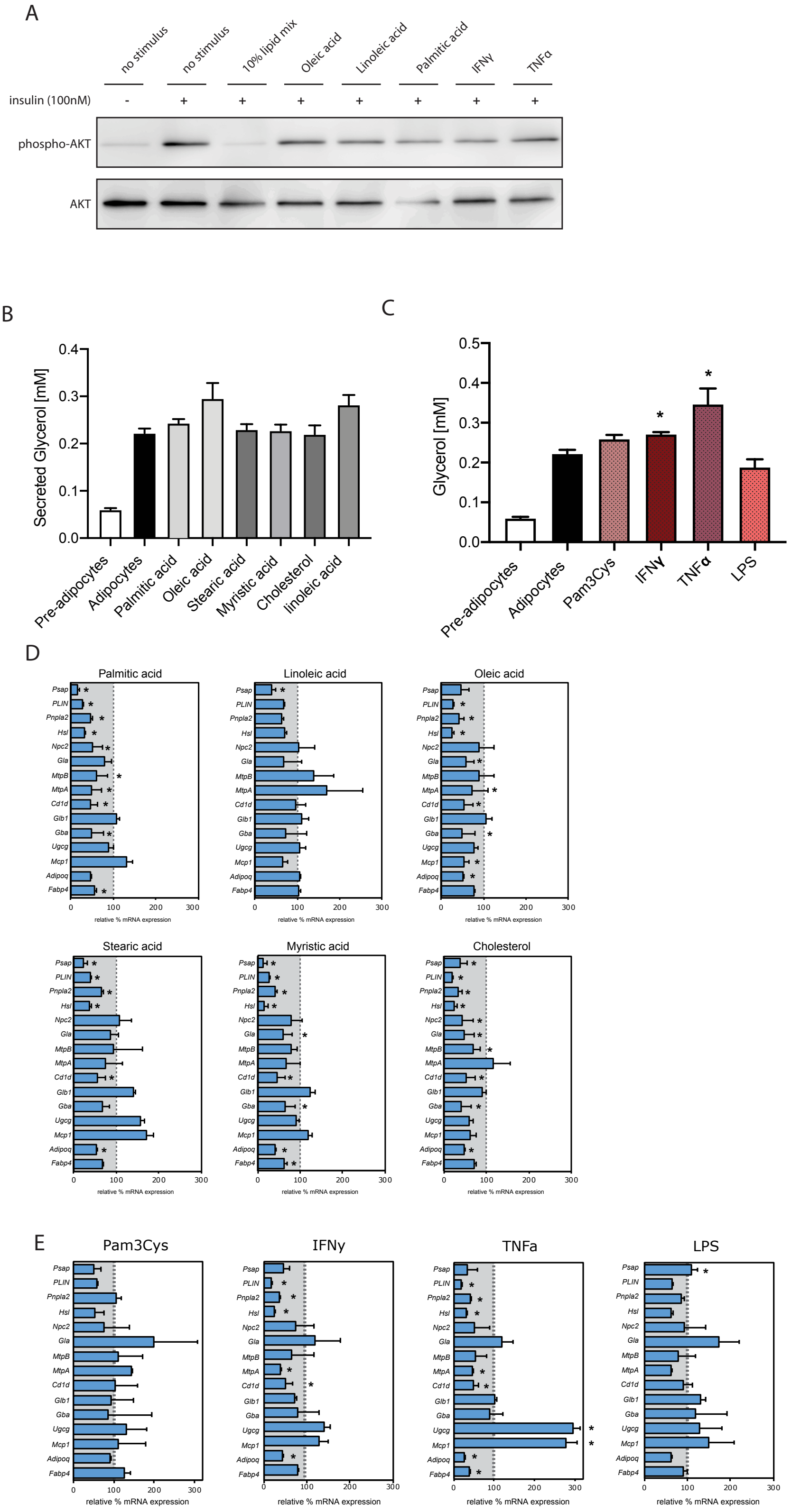

SUPPLEMENTARY FIGURE 2

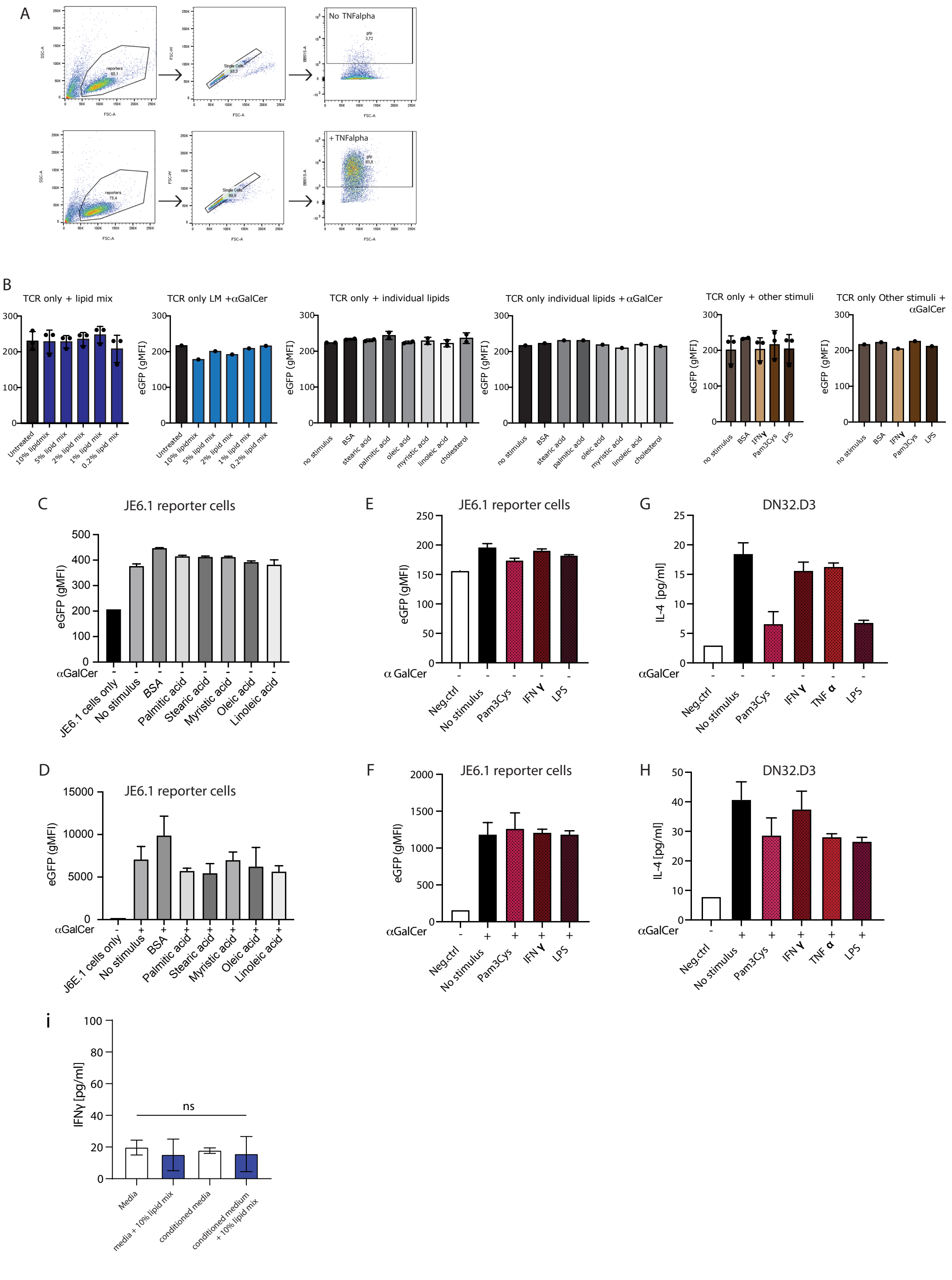

SUPPLEMENTARY FIGURE 3

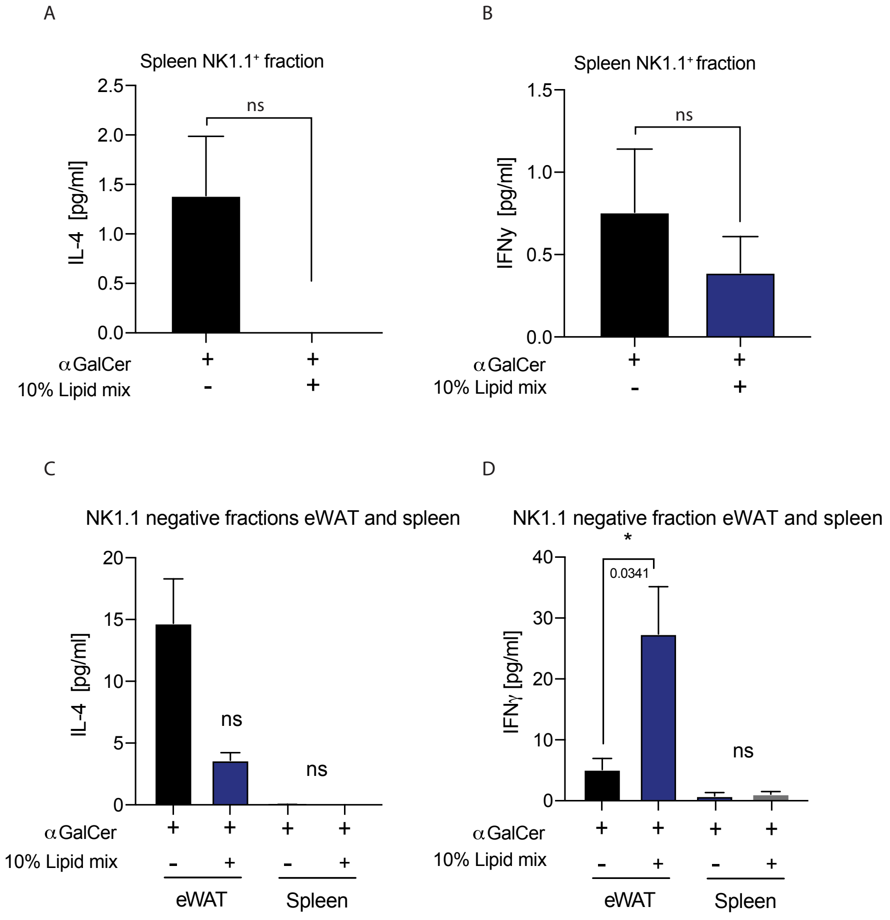

SUPPLEMENTARY TABLE 1

Supplementary Table 1, primers used for qPCR analysis

Name	Primer Forward	Primer Reverse
36b4	ATGGGTACAAGCGCGTCCTG	GCCTTGACCTTTTCAGTAAG
Fabp4	CGCAGACGACAGGAAGGT	TTCCATCCCACTTCTGCAC
Cd1d	CTGTCTGCGGGCTGTGAAAT	TCCCCAGAATCTCACGACATATT
Ugcg	TGCATGCTACACTTTCCTCTCCGT	AGCATCAGATGGATAACACGCCCT
Npc2	AATTGGGGCCACTTTTATCC	GCTCCGGATTCCAATACAAA
Mcp1	CCCAATGAGTAGGCTGGAGA	TCTGGACCCATTCCTTCTTG
Glb1	TCAAGGATGGACAGCCATTCCGAT	TCAGCCCAGCCATCTTCATCTTCA
α-*galactosidase*	CAGGGACGATGGTGATTTCT	GAAGCCAAACCTCTCAACCA
Psap	AGCAGAGGCAAAAACTTCCA	CAAGCTAGAGGTATCATGCTGAG
Gba	TTCACCAGACCTGGGCCAATTACT	TGCTGTAGGTTCATTCTCCGCTGT
Mtpa	AGCTGGCGTCCATCTGATAC	CCTGGGTCAAGAGGAAATGA
Mtpb	CCTGGGTCAAGAGGAAATGA	AGCTGGCGTCCATCTGATAC
Adipoq	GCAGAGATGGCACTCCTGGA	CCCTTCAGCTCCTGTCATTCC
Plin	CAAGCACCTCTGACAAGGTTC	GTTGGCGGCATATTCTGCTG
Pnpla2	GGAACCAAAGGACCTGATGA	GCTCTTTCATCCACCGGATA
Hsl	GAGCGCTGGAGGAGTGTTTT	TGATGCAGAGATTCCCACCTG

Acknowledgement:

This work was supported by funding from the European Union's Horizon 2020 Marie Skłodowska-Curie Innovative Training Network, TRAIN (project no. 721532)

TRAIN Consortium Authorship:

**Surname**	**First Name**	**Institution**
Bhutia	Kunzangla	Instituto de Investigacion Santaria, Hospital Clinico SanCarlos
Brouard	Sophie	University of Nantes
Barril	Xavier	University of Barcelona
Carracedo	Arkaitz	Asociación Centro de Investigación Cooperativa en Biociencias
Castillo Lluva	Sonia	Instituto de Investigacion Santaria, Hospital Clinico SanCarlos
Danger	Richard A.E.D	University of Nantes
Day	Jack	Instituto de Investigacion Santaria, Hospital Clinico SanCarlos
Deshmukh	Sumeet R	The University of Sheffield
Feseha	Yodit	University of Nantes
Francis	Sheila	The University of Sheffield
Grzesik	Dominika J	William Harvey Research Institute, Queen Mary University, London
Hernandez Quiles	Miguel	Universitair Medisch Centrum Utrecht
Kalkhoven	Eric	Universitair Medisch Centrum Utrecht
Kiss-Toth	Endre	The University of Sheffield

Linford	Adam J	Institute for Diabetes and Cancer (IDC), Helmholtz Zentrum München
Martinez Campesino	Laura	The University of Sheffield
Metherall	Louise A	William Harvey Research Institute, Queen Mary University, London
Morris	Imogen	Universitair Medisch Centrum Utrecht
Niespolo	Chiara	The University of Sheffield
Pellegata	Natalia S	Institute for Diabetes and Cancer (IDC), Helmholtz Zentrum München
Ruiz Cantos	Miriam	William Harvey Research Institute, Queen Mary University, London
Salamanca Viloria	Juan	University of Barcelona
Satam	Swapna S	Institute for Diabetes and Cancer (IDC), Helmholtz Zentrum München
Scheideler	Marcel J.C.	Institute for Diabetes and Cancer (IDC), Helmholtz Zentrum München
Shahrouzi	Parastoo	Asociación Centro de Investigación Cooperativa en Biociencias
Shologu	Ziyanda	Universidade da Beira Interior
Shoulders	Carol C	William Harvey Research Institute, Queen Mary University, London
Sudbery	Ian	The University of Sheffield
Socorro	Silvia	Universidade da Beira Interior

Velasco	Guillermo	Instituto de Investigacion Santaria, Hospital Clinico SanCarlos
Villacanas Perez	Oscar	MindtheByte Ltd
Wilson	Heather	The University of Sheffield
