# Supplementary figures and images for: Cytokine Output of Adipocyte-iNKT Cell Interplay Is Skewed by a Lipid-Rich Microenvironment
Source: Front Endocrinol (Lausanne). 2020 Jul 31;11:479. doi: 10.3389/fendo.2020.00479 (PMC7412741; doi:10.3389/fendo.2020.00479)

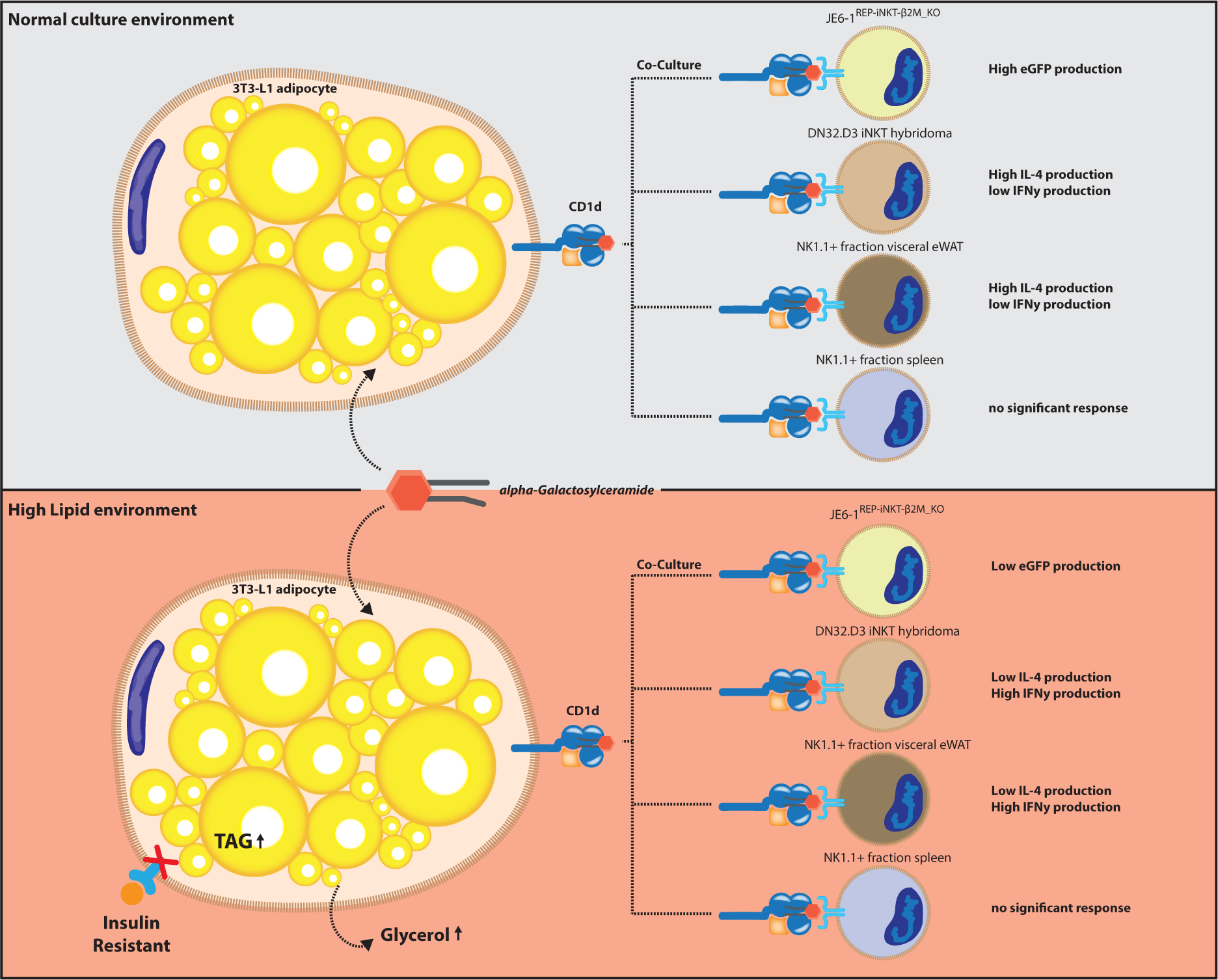

Supplement: Supplementary file 2 [file Image_1.TIF]
